# Utility of photoacoustic patterns in intra-operative margin assessment of breast cancer post neoadjuvant chemotherapy

**DOI:** 10.1016/j.pacs.2025.100701

**Published:** 2025-02-23

**Authors:** Yonggeng Goh, Ghayathri Balasundaram, Hui Min Tan, Thomas Choudary Putti, Renzhe Bi, Mikael Hartman, Shaik Ahmad Buhari, Celene Wei Qi Ng, Su Ann Lui, Serene Si Ning Goh, Wei Qi Leong, Eric Fang, Swee Tian Quek, Malini Olivo

**Affiliations:** a Department of Diagnostic Imaging, National University Hospital, 5 Lower Kent Ridge Road, Singapore 119074, Singapore; b A⁎STAR Skin Research Labs, Agency for Science, Technology and Research (A⁎STAR), 31 Biopolis Way, #07-01, Nanos 138669, Singapore; c Department of Pathology, National University Hospital, 5 Lower Kent Ridge Road, Singapore 119074, Singapore; d Department of Breast Surgery, National University Hospital, 5 Lower Kent Ridge Road, Singapore 119074, Singapore

**Keywords:** Ultrasound guided photoacoustics, Intra-operative margin assessment, Breast cancer, Neoadjuvant chemotherapy, Collagen, Tissue chromophores

## Abstract

**Purpose:**

To evaluate the feasibility and accuracy of ultrasound-guided photoacoustic tomography (US-PA) for intraoperative margin assessment in breast-conserving surgery (BCS) following neoadjuvant chemotherapy (NACT).

**Methods:**

This study, approved by the local Institutional Review Board, included 21 women with histologically confirmed breast cancer referred for BCS post-NACT. Data from 4 participants were used for training while 17 participants were analyzed. US-PA imaging was performed using the MSOT inVision 512-ECHO system, capturing chromophores like lipids, collagen, and hemoglobin up to a 5 mm depth. Imaging results were compared to histopathological findings, and diagnostic accuracy was calculated.

**Results:**

US-PA imaging demonstrated a high diagnostic accuracy of 89.0 %, with a sensitivity and negative predictive value (NPV) of 100 %, specificity of 86.9 %, and positive predictive value (PPV) of 59.4 %. Excellent inter-observer agreement (kappa = 1) was observed. No laser-induced tissue damage was noted. The average scan time per specimen was approximately 20 minutes. False positives (n = 11) were primarily due to post-therapy fibrotic changes and extremely close tumor extensions (<2 mm).

**Conclusion:**

US-PA provided clear visualization of tissue components, accurately correlating with histopathology. The method's high NPV minimizes the risk of re-operations and locoregional recurrence. Although the PPV was lower, it did not impact clinical management as surgeons typically excise wider margins in such cases. The study highlighted US-PA’s potential as a promising tool for intraoperative margin assessment in BCS post-NACT, offering a rapid, accurate, and safe method. Further studies with larger sample sizes are needed to confirm these findings and enhance quantitative assessment methods.

## 1 Introduction

Neoadjuvant chemotherapy (NACT) has become a mainstay in the treatment of locally advanced breast cancer [1], [2], [3]. It allows downstaging of tumours for breast-conserving surgery (BCS) with similar long-term oncologic outcomes as compared to mastectomy [4], [5] However, this comes at a short-term expense of locoregional disease recurrence risk [6], which can be reduced by obtaining clear/negative margins during BCS (i.e. complete tumour excision). It is hence imperative to have an accurate intra-operative margin assessment tool during BCS for patients who underwent NACT.

While the surgeons have a suite of intraoperative margin assessment tools to tap on to, the evidence of their clinical utility and practicality varies a lot. The wide variety of unpredictable changes after NACT often results in heterogeneous changes of the breast stroma which offers further challenges. Current established intra-operative margin assessment tools include clinical palpation, frozen section analysis (FSA), specimen radiography (SR) and intraoperative ultrasonography (US). Clinical palpation is particularly challenging in small tumors and dense breasts which is very common in Asians. FSA, the gold standard, is not practised in many institutions due to its high cost and long waiting-time. Furthermore, NACT is a significant predictor for false negative FSA [7]. SR has low sensitivity to detect positive margins after NACT [8]. US often reveals indistinct borders due to the heterogeneous changes in the breast stroma resulting from NACT [3], [9]. In addition, scattered foci of residual tumor may be present within the tumor bed, which are too small to be detected by US or through clinical inspection and examination of the excised breast tissue [10], [11]. To address these limitations, several intraoperative tools like optical spectroscopy, micro-CT and MarginProbe (reviewed in [12]) are developed and being validated in the recent years to see if they can challenge the diagnostic accuracy of the existing tools but at improved speed and reasonable cost. Ultrasound guided photoacoustics (US-PA) is one such modality that is being recently evaluated for breast cancer margin assessment [12], [13], [14], [15].

Photoacoustic Tomography (PA) is a hybrid optical imaging modality, which provides endogenous differentiation of imaged tissue contents such as hemoglobin, lipids, collagen at a greater depth than many other optical imaging modalities and hence is emerging as a promising preclinical & clinical imaging modality for various disease conditions [15], [16], [17] including breast cancer [18], [19], [20], [21]. When fused with US, PA could potentially resolve the heterogeneous changes on US by providing additional biochemical data (e.g. collagen from post NACT collagenization and fibrosis) which could improve diagnostic confidence [13], [14]. The greater depth of penetration offered by this modality compared to other optical spectroscopy methods or MarginProbe gives the technology an edge not just for surgical margin assessment but also for characterisation of lesion or improving the diagnostic confidence of the same [22]. Herein, the authors seek to identify optoacoustic signatures/pattern of breast tissue post NACT in this study, and to investigate its feasibility as biomarkers/prediction for margin assessment in the intra-operative setting.

## 2 Materials and methods

### 2.1 Study population

The study received approval from the local Institutional Review Board. From August 2020 to Jan 2022, we prospectively screened women over 18 years old with histologically confirmed breast cancer who were referred to the Department of Surgery, National University Hospital, Singapore, for breast-conserving surgery (BCS) after neoadjuvant chemotherapy (NACT). Patients who underwent total mastectomy post-NACT due to poor response to the therapy or non-conservable extent of breast disease were deemed unsuitable for surgery (e.g., due to distant metastases) and were excluded as BCS was not part of the routine clinical workflow for such cases. After obtaining written informed consent, 21 women were recruited. The data from the first four participants were allocated for training, while data from the remaining 17 were used for analysis.

### 2.2 Equipment and imaging protocol

The excised breast tissue following lumpectomy was oriented using silk stitches by surgeons, as depicted in Figure S1. Subsequently, the tissue underwent a saline rinse to eliminate surface blood. All specimens for this ex vivo investigation were collected within 10 minutes post-surgery from the operating theatre and promptly subjected to imaging to retain blood signals.

Detailed imaging protocol and histology analysis is provided in SI. Briefly, for ultrasound-photoacoustic (US-PA) imaging, we utilized the MSOT inVision 512-ECHO system (iThera Medical GmbH, Munich, Germany), along with a specially designed handheld two-dimensional (2D) optoacoustic probe. This probe possessed an arc-shaped array of 256 detector elements with 125º angular coverage, arranged on a spherical surface (radius 40 mm) and operated at a central frequency of 5 MHz. This set up, like other photoacoustic imaging platforms, faced depth penetration limitations due to light scattering and absorption in biological tissues. While it can image a few millimeters to a few centimeters deep, depending on tissue type and optical properties, in lumpectomy specimens, we noted that blood signals could be detected up to 7 mm and lipids up to 3 mm [15]. Its effectiveness diminished in deeper tissues, particularly, the inside of the tumor was underrepresented while the rim of the tumor was adequately represented [15]. The 2D probe offers an in-plane resolution of approximately 200 μm near the center of rotation, remaining stable within ± 10 mm [23].

To facilitate imaging, the specimen was positioned on a platform allowing for multidirectional movements—horizontal, vertical, and rotational. For imaging the flip side, the specimen was manually flipped. Scanning commenced by positioning the probe on a computer-controlled stage, starting from the thickest part of the specimen and traversing it horizontally and vertically (placed on a silicone bed within an imaging chamber) through heavy water (D2O) to ensure optimal acoustic coupling, as shown in Figure S1. Depending on size, the probe covered the entire specimen in increments of 1 mm.

Light for imaging was delivered via a fiber optic bundle integrated into the probe, employing a wavelength-tunable optical parametric oscillator with selectable wavelengths ranging from 660 nm to 1300 nm, at a repetition rate of 10 Hz and per-pulse energy of 80 mJ (at 730 nm). Various wavelengths (ranging from 700 nm to 1100 nm) were utilized to capture acoustic data from light-absorbing chromophores present in breast tissue, including Hb, HbO_2_, lipid, and collagen. The applied fluence remained below 20 mJ/cm^2^, adhering to the safety limit for NIR nanosecond lasers at 10 Hz set by the American National Standards Institute. Real-time images generated by the back-projection algorithm were displayed during data acquisition.

Following US-PA imaging, specimens were immersed in formalin and transported to the laboratory for subsequent histopathological examination.

### 2.3 Histopathological examination and juxtaposition with PA imaging

The plane of scan acquisition was conveyed to the pathologist, who sectioned the specimen accordingly to ensure accurate US-PA and histologic correlation. After post-processing, the radiologist, PA scientist and pathologist would gather in a multi-disciplinary meeting to ascertain PA-pathology correlation by identifying the index/suspicious lesion and its relationship with adjacent margins. For patients that exhibited completed response, the clip that was in the tumor was identified as the center of lesion while for patients with nil/partial response, the residual tumor was identified as the region of interest.

### 2.4 Image reconstruction and analysis

Offline reconstruction of images acquired at each wavelength was conducted using default settings (back-projection algorithm, cut-off frequencies at 0.5 khz to 6.5 Mhz) on ViewMSOT 3.8. Spectral unmixing was performed after discarding negative values (that arise from the use of linear array [24]) using the default linear regression algorithm in ViewMSOT 3.8 to differentiate between chromophores, including Hb, HbO_2_, lipids, and collagen. Collagen and lipid signals were unmixed based on the entire spectral range (700 – 1100 nm), while Hb and HbO_2_ signals were calculated from a sub-range (700–850 nm) for enhanced accuracy in unmixing due to lower water absorptivity at these wavelengths. Color maps were assigned to each chromophore: lipids were represented in green, collagen in magenta, and total hemoglobin (HbT= Hb+HbO_2_) in red. The images were transferred out of ViewMSOT and analysed on ImageJ. For the few negative pixels that may still persist following spectral unmixing, these values were further handled using ImageJ. Specifically, all pixel values less than zero were set to NaN (Not a Number), ensuring that the negative pixels do not influence the chromophore assessment. To optimize image processing and acquisition, US-PA images from the first participant in each category were evaluated by two unblinded readers, YG (a breast radiologist with 6 years of experience in photoacoustic imaging) and GB (with 8 years of experience in clinical photoacoustic imaging).

Tissue samples were categorized into two groups for analysis: group 1 consists of cases indicating pathological complete response (pCR) with no residual US mass, and group 2 consists of cases with residual US mass suggesting incomplete/partial response. Cases showing ill-defined heterogeneous changes post-NACT with no definite 3D dimensions of a mass-like lesion on US were classified into group 1, where the original tumor bed was identified by the localization/tissue marker clip inserted prior to NACT. In group 2, the tumor bed was identified by the presence of a residual mass with definite 3D dimensions.

Optoacoustic distribution maps were generated for each specimen, and the results were scrutinized to pinpoint optoacoustic patterns/signatures specific to each group. Initially, for training purposes, US-PA images from the initial 2 participants in each group (totaling 4 participants) were collectively evaluated by a panel of three unblinded readers: YG, a breast radiologist with 6 years of experience in photoacoustic imaging; GB, possessing 8 years of experience in clinical photoacoustic imaging; and MO, with 9 years of experience in clinical photoacoustic imaging. Following this, US-PA images for the remaining 17 participants were individually assessed by YG and GB, with any disparities resolved by MO, who was blinded to histopathologic findings.

In total, 102 margins were evaluated in excised specimens from the 17 study participants, with each specimen comprising 6 margins (anterior, posterior, superior, inferior, medial, and lateral). Presently, chromophore signals are subjected to visual and qualitative evaluation, though there are intentions to devise a quantitative assessment method in subsequent studies.

### 2.5 Statistical analysis

The results from margin assessments using ultrasound-photoacoustic (US-PA) imaging were compared to histopathologic findings in a categorical manner (i.e., positive/negative). Cross-tabulation was conducted for all assessed margins. The overall diagnostic accuracy, sensitivity, specificity, positive predictive value (PPV), and negative predictive value (NPV) of US-PA for margin diagnosis were calculated using SPSS software (Version 22; PASW Statistics, Chicago, Ill). Kappa statistics were determined to assess the overall agreement of US-PA with histopathology and to evaluate inter-observer variation in the assessment of US-PA images between YG and GB. This method was chosen as it is widely used for evaluating the reliability of categorical assessments and is particularly appropriate for our study, where multiple observers independently evaluated the same imaging data. Given the exploratory nature of this pilot proof-of-concept study, no sample size calculation was performed.

## 3 Results

### 3.1 Patient characteristics

Table 1 displays the baseline characteristics, histopathology of breast tumors, and the size of lumpectomy specimens from the 17 participants included in the analysis, with a mean age of 57.0 years ± 8.4.

**Table 1:** Baseline characteristics of patients (n = 17).

Parameter	Value
Participant Characteristics	
Age, years	
Mean	57.0
Range	37 – 73
Group 1 (complete response)	9/17 (52.9 %)
Group 2 (nil/partial response)	8/17 (47.1 %)
Breast density	
A: entirely fatty	0/17
B: scattered fibroglandular tissue	2/17
C: heterogeneously dense	15/17
D: extremely dense	4/17
Histology	
IDC (Invasive Ductal Carcinoma)	12/17 (70.5 %)
Combined IDC + DCIS (Ductal Carcinoma in-situ)	2/17 (11.8 %)
ILC (Invasive Lobular Carcinoma)	1/17 (5.9 %)
Others (i.e. Mixed IDC + Micropapillary carcinoma, Invasive mucinous carcinoma)	2/17 (11.8 %)
Tumor Markers	
ER+ PR+ HER−2-	5
ER+ PR- HER−2 +	1
ER- PR+ HER−2-	1
ER- PR- HER−2 +	2
ER- PR- HER−2- (triple negative)	5
ER+ PR+ HER−2 + (triple positive)	3

### 3.2 Photoacoustic patterns observed across the NACT specimens

The photoacoustic distribution maps of lipid, collagen and hemoglobin (Hb) from the specimens were acquired up to a depth of 5 mm (data not shown).

**Fig. 1:**
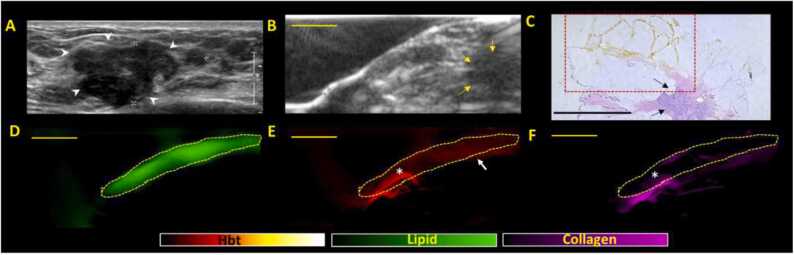
**A Representative NACT specimen showing continuous lipid signal, a pattern for negative margins on US-PA imaging.** (A) Clinical in vivo ultrasound image shows presence of an irregular hypoechoic mass (white arrow heads) in the left breast which was biopsied proven to be a grade 3 Invasive carcinoma (no special type). The patient subsequently underwent NACT. (B) e*x-vivo* US image of the surgically excised specimen showing a small residual hypoechoic mass (yellow arrows) which is in keeping with partial/incomplete response. (C) Haematoxylin and eosin (H&E) stained microscopic image of specimen showing residual tumor (black arrows) that is at least 3 mm away from all margins (boxed area corresponds to PA signals shown in (D-F). **D, E, F** shows the corresponding total lipid, hemoglobin, and collagen distribution maps generated from PA imaging. The presence of a continuous lipid signal (dotted area) in (D) is highly indicative of the presence of negative margins. The presence of some blood signals (white arrow) in (E) is normal and expected from post-surgical status/bleeding). The presence of high intensity blood and collagen signals (*) at the periphery of the excised tissue is due to orientation stitches (see Fig. S1). Scale bars: C, 10 mm, (B, D-F), 5 mm.

The first PA pattern identified was the presence of intense and continuous lipid signals along the margins. A representative NACT specimen with partial/incomplete treatment response showing this pattern is presented in Fig. 1. The continuous lipid pattern (Fig. 1D), noted at the margins, is highly indicative of negative/clear margins with sensitivity, specificity, PPV, and NPV of 100 %, 54.8 %, 51.7 % and 100 % respectively. In areas of lipid discontinuity/disruption, optoacoustic distribution maps invariably identified collagen and hemoglobin signals. Areas with no lipid, collagen or hemoglobin signals were constantly related to imaging artefacts (e.g., air bubbles).

The optoacoustic distribution maps for collagen and hemoglobin at the margins, which resulted in discontinuity of lipid layer, demonstrated three patterns at the specimen margins when compared to the tumor bed. These patterns are as described:

#### 3.2.1 Intensity of collagen signal at margins vs tumor bed

If collagen signals are present at the margins, its signal intensity is directly compared to the signal intensity of the tumor bed. Overall findings are considered positive/suspicious if the collagen signals at the margins are equal or more intense as compared to the tumor bed, as these collagen signals are postulated to represent direct/indirect spread of disease towards the margins. If the collagen signals are less intense than the tumor bed, these findings are more likely to represent normal breast tissue on histology.

#### 3.2.2 Intensity of hemoglobin signal at margins

After inspection of collagen signals at the margins and comparing its intensity against the tumor bed, the authors also evaluated the vascularity of these regions with the use of hemoglobin signals. If the hemoglobin signals are more intense compared to the tumor bed or its surroundings, findings would be deemed as positive/suspicious as tumour cells are known to demonstrate increased vascularity due to angiogenesis. Areas of increased hemoglobin signals with no corresponding collagen signals are often due to presence of hematoma/bruises which are normal/expected from surgeries.

#### 3.2.3 Connectivity of collagen and hemoglobin signal at margins to the tumor bed

After inspection of collagen and hemoglobin signals at the margins as described in (A) and (B), the authors observed another optoacoustic pattern which is connectivity of these signals towards the tumor bed. If the collagen/hemoglobin signals show a direct connection towards the tumor bed, findings are deemed to be suspicious and worrisome for direct spread of disease.

### 3.3 Overall interpretation

The overall interpretation of margins depends on the permutation of the above three categories. The categories above should not be read/interpreted in isolation as these factors are interconnected. For example, areas of abnormal collagen intensity at the margins may be worrisome for positive margins. However, in the absence of increased vascularity or connectivity towards the tumor bed, these areas of abnormal collagen could represent other collagenous breast conditions such as scarring/fibrosis which is an expected finding after NACT.

**Fig. 2:**
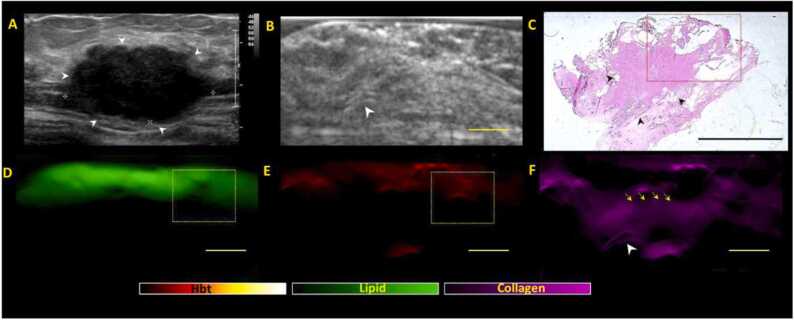
**Positive margin patterns on US-PA images in a representative Group 1 specimen (no residual lesion on imaging post NACT, suggestive of pathological complete response (pCR).** (A) Clinical in-vivo ultrasound pre NACT shows the presence of a 3.5 cm irregular hypoechoic mass (white arrow heads) in the left breast which was biopsied proven to be a grade 2 invasive carcinoma (no special type). Patient subsequently underwent NACT (B) ex-vivo US post NACT shows no residual lesion upon surgical excision, highly suggestive of pCR. There is a faint echogenic clip noted (arrowhead) in keeping with the tumour bed location. (C) Haematoxylin and eosin (H&E) stained microscopic image of specimen shows areas of fibrosis on low power view (black arrowheads), but there was residual high-grade DCIS (Ductal carcinoma in-situ) extending towards the posterior margins (red dotted box). The DCIS was 1.5 mm away from the posterior margins and would be deemed as positive margins (<2 mm for DCIS). (D, E, F) shows the corresponding lipid, total hemoglobin (Hbt) and collagen distribution maps generated from PA imaging. There is focal disruption of continuous lipid signal (yellow dotted box) in (D). There is presence if mildly increased Hbt (E) and collagen (F) signals at the area of lipid disruption. Of note, the collagen shows direct connectivity (yellow arrows) to the tumor bed/clip (arrowhead). The presence of all 3 PA imaging criteria were met and findings are suspicious for focal involvement of the posterior margins, concurring with histological findings. Scale bars: C, 10 mm, (B, D-F), 5 mm.

Hence, the interpretation of margins would require permutation of the three optoacoustic patterns as described above (A-C):

For group 1 (no residual lesion suggestive of pCR), the index tumour has demonstrated complete response to NACT. The threshold for considering positive margins would hence be higher. For group 1, if the lipid layer is disrupted, a margin would only be positive on US-PA if all 3 criteria (A-C) are met (Fig. 2). Margins would be classified as negative if ≤ 2 criteria from (A-C) are met.

**Fig. 3:**
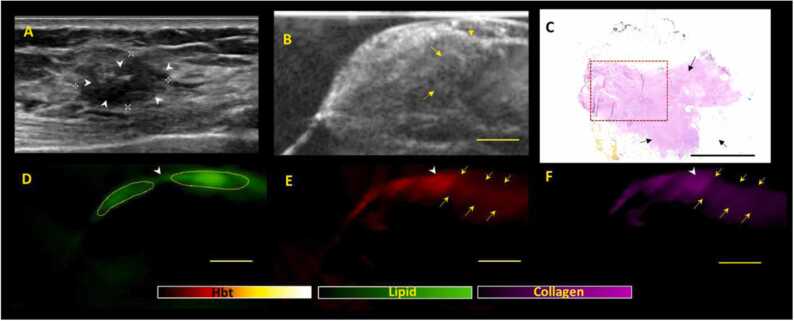
***Positive margin patterns on US-PA images in a representative NACT specimen showing residual lesion post NACT, suggestive of partial/incomplete response (Group 2).*** (A) Clinical in-vivo ultrasound pre NACT shows the presence of a 1.5 cm irregular hypoechoic mass (white arrow heads) in the left breast which was biopsied proven to be a grade 3 invasive carcinoma (no special type) with associated ductal carcinoma in-situ. The patient subsequently underwent NACT (B) ex-vivo US post NACT shows presence of residual lesion (yellow arrows) upon surgical excision, in keeping with partial/incomplete response. There is seemingly normal heterogeneous breast tissue between the residual tumour and the imaged inferior margin. (C) Haematoxylin and eosin (H&E) stained microscopic image of specimen shows residual tumor (black arrows) with some response to presurgical therapy. The tumor bed extends up to all margins grossly with DCIS extending very close to the inferior margin (1 mm) (red dotted box). The tumor cells (both invasive and in-situ) also extend very close to (1 mm or less than 1 mm away from) several other margins (anterior, superior, medial and lateral) microscopically. (D, E, F) shows the corresponding lipid, total hemoglobin and collagen distribution maps generated from PA imaging. There is thinning/disruption of the lipid layer (D) noted (white arrowheads) at the superior margin. The area of disruption is noted to contain high intensities of hemoglobin (E) and collagen (F) (white arrowheads) with direct connectivity to the tumor bed (yellow arrows). The presence of all 3 PA imaging criteria for positive margins were met and hence, PA imaging findings are highly indicative of positive margins. Scale bars: C, 10 mm, (B, D-F), 5 mm.

**Fig. 4:**
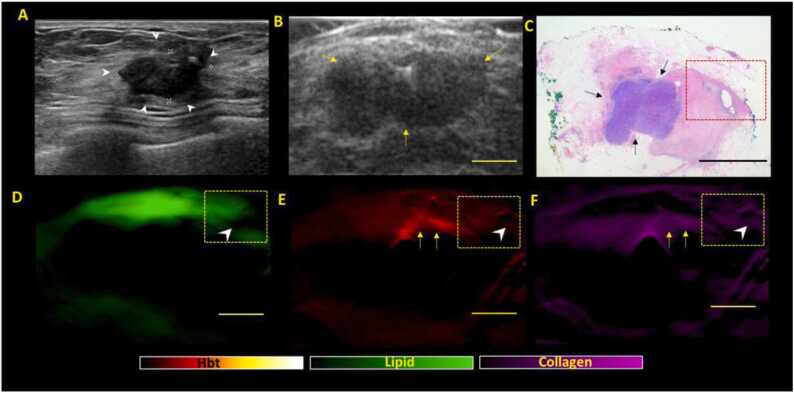
***Negative margin patterns on US-PA images in a representative NACT specimen showing residual lesion post NACT, suggestive of partial/incomplete response (Group 2).*****(A)** Clinical in-vivo ultrasound pre NACT shows the presence of a 2.0 cm irregular hypoechoic mass (white arrow heads) in the right breast which was biopsied proven to be a grade 3 invasive carcinoma (no special type). The patient subsequently underwent NACT (B) e*x-vivo* US post NACT shows presence of residual lesion (yellow arrows) upon surgical excision, in keeping with partial/incomplete response. (C) Haematoxylin and eosin (H&E) stained microscopic image of specimen shows residual tumor (black arrows) that is away from the margins, with surrounding fibrosis (tumor bed) extending closer to the margins (boxed area). (D, E, F) shows the corresponding lipid, total hemoglobin and collagen distribution maps generated from PA imaging. There is thinning/disruption of the lipid layer (D) noted (white arrowhead) along the lateral margins of the specimen. The area of disruption is noted to contain collagen (F) which is directly connected to the tumor bed (arrowheads). However, this region of collagen signal intensity is not equal or higher than the tumour bed (yellow arrows) and this region does not show increased vascularity as compared to the tumor bed. Hence, only 1 imaging PA criteria was met (collagen connectivity to tumour bed) and findings are supportive of negative margins. Scale bars: C, 10 mm, (B, D-F), 5 mm.

For group 2 (with residual US mass suggestive of incomplete/partial response), the threshold for positive margins would be slightly lower as these lesions are not complete responders which raise the possibility of residual cancer cells. For group 2, if the lipid layer is disrupted, a margin would be positive if ≥ 2 criteria from (A-C) are met (Fig. 3). Margins would be classified as negative if < 2 criteria from (A-C) are met (Fig. 4).

**Fig. 5:**
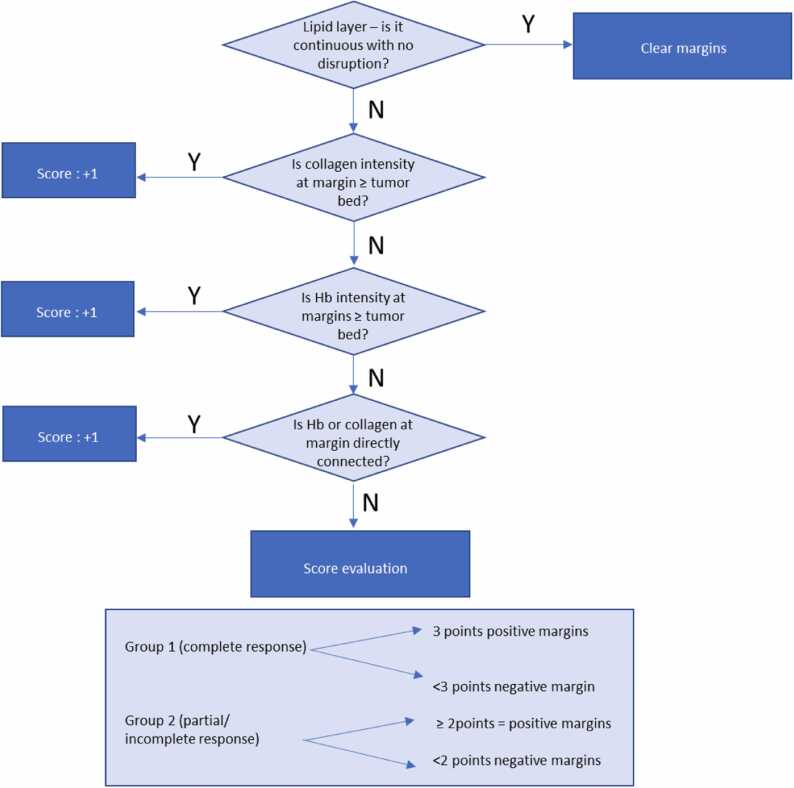
Decision tree on assessment of surgical margins based on photoacoustic patterns.

Overall findings are summarized in the flowchart below (Fig. 5).

### 3.4 Diagnostic accuracy of US-PA

Aligned with the standards established by the Society of Surgical Oncology, which define acceptable margins as "no ink on tumor" for invasive ductal carcinoma (IDC) and a 2-mm negative margin for ductal carcinoma in situ (DCIS), we classified margins as involved/positive if there was ≤ 2 mm of normal breast tissue observed between the tumor and the excised breast tissue margin of concern.

Among the 102 margins evaluated from 17 participants, two margins were excluded due to heavy staining by patent blue, rendering the photoacoustic (PA) image uninterpretable. The analysis was conducted on the remaining 100 margins, categorized according to the respective diagnostic criteria (Group 1: 54 margins, Group 2: 46 margins).

For group 1, the diagnostic accuracy of US-PA showed a diagnostic accuracy of 98.1 % (sensitivity, specificity, positive predictive value (PPV) and negative predictive value (NPV) of 100 %, 98.1 %, 50.0 % and 100 % respectively).

For group 2, the diagnostic accuracy of US-PA showed a diagnostic accuracy of 78.3 % (sensitivity, specificity, positive predictive value (PPV) and negative predictive value (NPV) of 100 %, 67.7 %, 60.0 % and 100 % respectively).

Overall, US-PA achieved a combined diagnostic accuracy of 89.0 % (Group 1 + 2) with sensitivity, specificity, PPV and NPV of 100 %, 86.9 %, 59.3 % and 100 % respectively. Interpretation of the relationship of these 3 criteria with positivity/negativity of margins is summarized in Table 2.

**Table 2:** Interpretation of the relationship of these 3 criteria with positivity/negativity of margins.

**GROUP 1 (54 margins)**		HISTOLOGY	
		POSITIVE	NEGATIVE
US-PA	POSITIVE	1	1
	NEGATIVE	0	52
**GROUP 2 (46 margins, 2 excluded)**		HISTOLOGY	
		POSITIVE	NEGATIVE
US-PA	POSITIVE	15	10
	NEGATIVE	0	21
**COMBINED (GROUP 1 + 2)**		HISTOLOGY	
		POSITIVE	NEGATIVE
US-PA	POSITIVE	16	11
	NEGATIVE	0	73
OVERALL RESULTS (100 MARGINS)			
	GROUP 1	GROUP 2	COMBINED
Sensitivity	100.0 %	100.0 %	100.0 %
Specificity	98.1 %	67.7 %	86.9 %
PPV	50.0 %	60.0 %	59.3 %
NPV	100.0 %	100.0 %	100.0 %
Accuracy	98.1 %	78.3 %	89.0 %

No interpretive discrepancies were observed between the two readers (YG and GB) for the remaining 100 margins assessed, indicating excellent inter-observer agreement (inter-observer variability = 1, kappa = 1). Histopathological examination revealed no evidence of laser-induced tissue damage to the excised specimen. The average scan time for each specimen in our study was approximately 20 minutes.

### 3.5 False positives

**Fig. 6:**
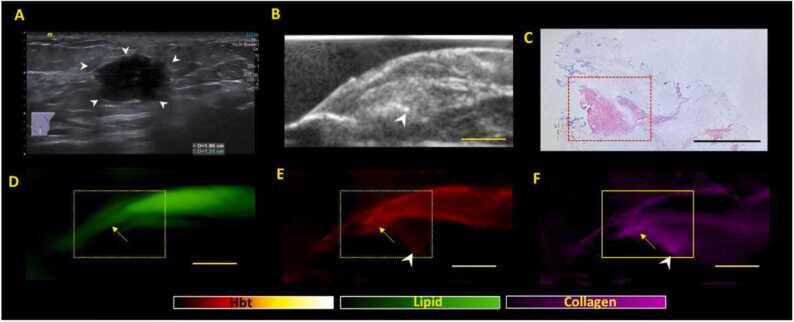
***False positive margin on US-PA images in a representative NACT specimen showing no residual lesion post NACT on imaging, suggestive of pathological complete response (pCR) (Group 1).*****(A)** Clinical in-vivo ultrasound pre NACT shows the presence of a 2.0 cm irregular hypoechoic mass (white arrow heads) in the left breast which was biopsied proven to be a grade 3 invasive carcinoma (no special type). The patient subsequently underwent NACT (B) e*x-vivo* US post NACT shows no residual lesion upon surgical excision, highly suggestive of pCR. An echogenic clip (white arrowhead) is seen, representative of the tumor bed location. (C) Haematoxylin and eosin (H&E) stained microscopic image of specimen shows no residual tumor, consistent with complete response to presurgical therapy. Residual tumor bed was identified near the lateral margin (boxed area). (C, E, F) shows the corresponding lipid, total hemoglobin and collagen distribution maps generated from PA imaging. There is thinning of the lipid layer along the lateral margin of the excised tissue, interspersed by dark linear bands (dotted box). The region of lipid thinning is occupied by increased signals of collagen and hemoglobin (yellow arrow heads). The signals of these chromophores show connectivity to the tumor bed (white arrowhead) and their intensities are equal or raised as compared to the tumor bed. All 3 PA imaging criteria for positive margins were met and hence, PA imaging findings would be worrisome for positive margins. However, there was no residual tumor cells noted on histology. This is an example of a false positive finding due to fibrotic changes post NACT abutting the surgical margins. Scale bars: C, 10 mm, (B, D-F), 5 mm.

**Fig. 7:**
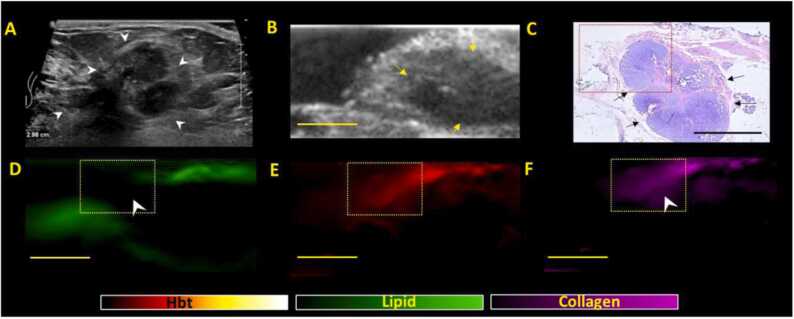
**False positive margin on US-PA images in a representative NACT specimen showing residual lesion post NACT, suggestive of partial/incomplete response (Group 2). (A)** Clinical in-vivo ultrasound pre NACT shows the presence of a 3.0 cm lobulated heterogeneous mass (white arrowheads) in the left breast which was biopsied proven to be a grade 2 invasive carcinoma (no special type). The patient subsequently underwent NACT. (B) e*x-vivo* US post NACT shows presence of residual mass (yellow arrows) upon surgical excision, in keeping with partial/incomplete response. (C) Haematoxylin and eosin (H&E) stained microscopic image of specimen shows residual tumor (black arrows) that is close to but not involving the posterior (1 mm away) and inferior (2 mm away) margins (red dotted box). The tumor also extends close to but not involving other margins (2 mm clearance from medial and anterior margins, not shown here). (D, E, F) shows the corresponding lipid, total hemoglobin and collagen distribution maps generated from PA imaging. There is disruption of the lipid layer (C) noted (white arrowhead in the yellow box) along the medial margins of the specimen. The area of disruption is noted to contain collagen which is directly connected to the tumor bed. The collagen and hemoglobin signal intensities are much higher than the tumour bed. In this scenario, all 3 PA imaging criteria were met, and findings are worrisome for positive margins. However, there was at least 1 mm of margin clearance between the tumor and the margins on histology. This is hence an example of false positive margins due to extremely close distance between the tumour cells and the margin. Scale bars: C, 10 mm, (B, D-F), 5 mm.

There were total of 11 false positive margins in this study (1 in group 1 and 10 in group 2). For group 1 where there was complete response, the false positive margin was secondary to the presence of post-therapy fibrotic changes at the margin (Fig. 6). There were no residual cancer cells at the area of fibrosis on histopathology. For group 2, 7/10 (70 %) of the false positive cases were a result of extremely close extension of tumor cells to the margin. The distances range from 1 to 2 mm with an average of 1.3 mm (Fig. 7). The other 3/10 (30 %) of the false positives were similar to Group 1 where there was presence of post-therapy fibrotic changes at the margin.

## 4 Discussion

In this study, we evaluated the feasibility and accuracy of ultrasound-guided photoacoustic tomography (US-PA) in assessing the margins of breast-conserving surgery (BCS) specimens following neoadjuvant chemotherapy (NACT). This approach has become increasingly pertinent due to the growing use of NACT, which, although initially reserved for locally advanced breast cancers (LABCs) [3], is now recommended as the preferred treatment for HER2-positive (HER2 +) and triple-negative breast cancer (TNBC) [25], [26], even in early-stage cases where tumors are larger than 1.5 cm, as per guidelines from the American Society of Clinical Oncology (ASCO) and the National Comprehensive Cancer Network (NCCN). NACT not only reduces tumor size but also improves cosmetic outcomes and lowers the risk of postoperative complications, such as lymphedema [4], [5]. Given the widespread adoption of NACT, reliable tools for accurate margin assessment are critical in reducing the need for repeat surgeries. Our findings demonstrate that US-PA effectively visualized the distribution of lipids, collagen, and hemoglobin up to a depth of 5 mm, with a margin assessment accuracy of 89.0 % compared to histopathology. While we began evaluating tumor margins using two biomarkers initially: lipids and hemoglobin [15] due to their relevance in distinguishing tumor boundaries and the availability of clear photoacoustic signals for these chromophores. we realised that in dense Asian breasts, the presence of fibroglandular tissues at the margins led to similar patterns like that of positive margins [15]. The wide variety of unpredictable changes after NACT often results in heterogeneous changes of the breast stroma due to fibrosis. As the study progressed, the photoacoustic profile of collagen was successfully established using multispectral photoacoustic imaging and collagen was used as an additional biomarker in tumor margin assessment [13].

In particular, the high NPV of 100 % makes US-PA a potential intra-operative tool for assessment of margins in BCS. The assurance of a negative margin obtained reduces the risks of re-operations and locoregional recurrence risks. The PPV is however relatively low at 59.4 % in our study. The majority (7/11) of these can be explained by extremely close tumour margins (<2 mm). Close margins appear as positive margins in photoacoustic imaging due to the system's sensitivity in detecting subtle changes in tissue composition. When tumor cells are located very close to the margin (e.g., within 1–2 mm), the proximity of these cells may result in signal overlap with normal tissue or tissue affected by fibrotic or inflammatory changes. This overlap can produce a signal that mimics the appearance of a positive tumor margin, even though the tumor may not have fully extended into that region. The spatial resolution of the system (∼200 μm) limits its ability to distinguish between tumor and surrounding non-malignant tissues in very close proximity, resulting in a false positive. Additionally, in post-treatment settings, where scarring or fibrotic tissue is present, photoacoustic signals may be further confounded by changes in tissue density and composition, making it more challenging to accurately delineate tumor boundaries. These factors contribute to the appearance of close margins as positive margins in photoacoustic imaging.

From a clinical perspective, this is understandable and would result in no change in clinical management. This is because surgeons would err on the side of caution and obtain wider margins in these instances to reduce risks of locoregional recurrence. For the remaining 4/11, the false positive cases were secondary to post chemotherapy fibrotic changes at the margins which mimicked the features of positive margins on US-PA. As these entities share similar US-PA features qualitatively, future quantitative studies which measure the intensity of chromophores may be helpful in differentiation between true and false positive margins.

While it may be of interest to readers to compare the US-PA results with other studies, there is limited evidence on how the different technologies assess surgical margins following NACT. In our literature search, specimen radiography is the only modality that has been tested for margin assessment on specimens following NACT and has been shown to have low sensitivity to detect positive margins after NACT [8]. For ultrasound or margin probe, there are no specific studies on the margin assessment accuracy numbers post NACT. Ultrasound in general, has sensitivity ranging from 59 % to 100 %, and the specificity varies between 81 % and 100 %, depending on factors such as the study design, the surgeon's experience, and the characteristics of the lesions being assessed [22], [23]. Margin Probe has reduced re-excision rates by more than 50 % with a higher sensitivity at the expense of decreased specificity [27]. It is important to note that these numbers may not apply to surgical margin assessment in specimens post NACT as it becomes harder to assess because of the variety of response/changes after NACT.

The unique US-PA patterns/signatures of margin assessment as mentioned above are first described in this paper. In our study, these novel US-PA patterns/signatures have also been shown to correlate accurately with histopathology. In addition, these optical signatures also coincide with other studies which investigated the tumor microenvironment/collagen compartments of breast cancer. For example, collagen connectivity between tumour and the margins was described as a feature in a study published by Gole et. al [28]. Gole et. al described the presence of long collagen fibres/bands around tumours which serve as a path for potential tumor cell migration or immune cell response. The authors believe these US-PA features may not only be useful in assessment of tumor margins but could also be helpful in future studies which aim to explore the tumor microenvironment of breast cancers.

The US-PA patterns/signatures were also easy to interpret. The inter-observer agreement was 1.0, showing high correlation between readers. Coupled with the clear optical contrast between different chromophores and the high sensitivity of US-PA to endogenous chromophores, US-PA is emerging as a promising tool due to easy discrimination. US-PA is also postulated to have a gentle learning curve as radiologists will be basing the primary investigation on the already familiar US. US-PA is label-free and does not rely on any exogeneous contrast agents which are often toxic. US-PA is safe as we found no histopathologic evidence of laser-induced damage to the scanned specimens.

Furthermore, US-PA imaging demonstrated efficiency with an average scan time of 20 minutes per specimen, followed by 5–10 minutes for post-processing using the current algorithm. Reviewing the chromophore distribution maps and comparing them with histopathology is a slightly more time-intensive step, requiring approximately 10–15 minutes per sample. Understanding that the total time required for specimen assessment, including imaging and decision-making, is critical for workflow efficiency and patient safety, we have identified several areas for improvement to significantly reduce the overall turnaround time – 1. Automated Sample Stage: We are developing an automated sample stage that can image all specimen margins without the need for manual repositioning. This improvement is anticipated to reduce image acquisition time by at least 25 %. 2. Algorithm Refinement and Automation: Optimization of the current post-processing algorithms is underway, with a focus on enhancing efficiency and incorporating automation to further streamline the workflow. 3. Decision Algorithm Using a Database: Over time, we have imaged a diverse range of breast cancer specimens across various subtypes, compiling a growing database of photoacoustic images and corresponding chromophore distribution maps. This database serves as the foundation for developing an automated decision algorithm capable of analyzing specimens, identifying key features, and assessing margin involvement. We are actively working towards streamlining the entire process by optimizing post-processing workflows and automating the review step to reduce the total turnaround time to under 30 minutes, making the technology viable for intraoperative use. This is a significant improvement compared to the extended duration typically required for histopathological examination of tissue specimens and remains well within the recommended cold ischemia time of one hour. This underscores the potential integration of US-PA into the intraoperative clinical setting, enabling prompt decisions regarding the necessity for tissue re-excision to ensure complete tumor excision. Such integration has the potential to mitigate the requirement for repeat surgical procedures, thereby reducing the risks associated with patient morbidity.

The technology’s potential for widespread adoption is promising due to the global availability of ultrasound systems. By integrating seamlessly with existing ultrasound equipment, it fits easily into current clinical workflows. This integration minimizes disruption and accelerates market entry, as it maintains a familiar workflow and layout. The combination of low initial investment, reduced need for follow-up surgeries, and decreased post-operative costs highlights the technology's cost-effectiveness. Despite minimal upfront costs, long-term savings from fewer reoperations, lower complication rates, and more efficient resource use can lead to substantial financial benefits for healthcare systems.

Nevertheless, this feasibility study is not without limitations. Firstly, the probe was only able to resolve lipid, collagen and blood signals up to a maximum depth of 5 mm (spatial resolution: 200 µm). These findings could be related to a lack of active circulation due to the ex-vivo nature of study. These findings could potentially affect the interpretation as one may not accurately depict the connection/collagen bands from the margins to the residual tumour in a large sample where the tumor is deep-seated. These findings could be improved by devising dedicated imaging probes for breast (e.g. dimensions of probe) or by modifying light delivery methods (e.g., illumination from multiple angles). For instance, while the arc-shaped transducer delivers superior photoacoustic image quality, it may slightly compromise the quality of ultrasound imaging compared to a linear array. To overcome this, a **hybrid-shaped transducer can be designed** by merging the benefits of both designs by incorporating lower center frequency elements to enhance photoacoustic imaging and higher center frequency elements to improve ultrasound image clarity [29]. By introducing multiangle illumination, the depth of penetration and image quality can be considerably increased [30]. Secondly, the imaging set up used in this study, like other photoacoustic imaging platforms, suffers from unknown light fluence in deep tissues for quantitative unmixing of molecules. While the proprietary software ViewMSOT 3.8 accounts for this issue to some extent by incorporating wavelength dependent attenuation, there is room for improvement which can be achieved by employing advanced spectral unmixing models like eigenspectral MSOT (eMSOT) [31] or Monte Carlo simulation based light propagation models in tissues [32]. Thirdly, the sample size (n = 17) for this study was very small, necessitating a larger cohort study to quantitatively corroborate our findings. Nevertheless, according to the authors’ knowledge, this study remains one of the first with the largest number of margins assessed (100 margins).

## 5 Conclusion

In conclusion, our findings demonstrate that the ultrasound-guided optoacoustic tomography (US-PA) probe provided comprehensive structural and functional insights into the margins of lumpectomy specimens, effectively delineating tissue chromophores. The sensitivity of US-PA imaging to collagen, hemoglobin, and lipid holds significant promise for intraoperative assessment of tumor margins in post-neoadjuvant chemotherapy (NACT) patients, offering a safe, rapid, and accurate approach. The strong agreement between US-PA analysis and histopathologic interpretation suggests its potential utility as a tool for intraoperative margin assessment.

## Funding source

1. 10.13039/501100001348Agency for Science, Technology and Research (A*STAR), for the intramural funding support, Central Research Fund (UIBR) 2021
2. 10.13039/501100001321National Research Foundation, Singapore funded Translational Biophotonics Innovation Platform IAF PP fund (H19H6a0025)
3. National Medical Research Council’s Clinician Scientist individual research grant- new investigator grant (CS-IRG-NIG).

## CRediT authorship contribution statement

**Putti Thomas Choudary:** Writing – review & editing, Resources, Investigation. **Olivo Malini:** Writing – review & editing, Supervision, Resources, Conceptualization. **Tan Huimin:** Writing – review & editing, Writing – original draft, Visualization, Methodology, Investigation. **Quek Swee Tian:** Writing – review & editing, Supervision, Resources. **Balasundaram Ghayathri:** Writing – review & editing, Writing – original draft, Visualization, Validation, Methodology, Investigation, Formal analysis. **Fang Eric:** Writing – review & editing, Resources. **Goh Yonggeng:** Writing – review & editing, Writing – original draft, Visualization, Validation, Resources, Investigation, Funding acquisition, Formal analysis, Conceptualization. **Buhari Shaik Ahmad:** Writing – review & editing, Resources. **Hartman Mikael:** Writing – review & editing, Resources. **Bi Renzhe:** Writing – review & editing, Resources, Methodology. **Leong Wei Qi:** Writing – review & editing, Resources. **Goh Serene Si Ning:** Writing – review & editing, Resources. **Lui Su Ann:** Writing – review & editing, Resources. **Ng Celene Wei Qi:** Writing – review & editing, Resources.

## Declaration of Competing Interest

None

## Data Availability

Data will be made available on request.

## References

[1] Kaufmann M., Morrow M., von Minckwitz G., Harris J.R. (2010 Mar). Locoregional treatment of primary breast cancer: consensus recommendations from an International Expert Panel. Cancer.

[2] Fernandes J., Sannachi L., Tran W.T., Koven A., Watkins E., Hadizad F. (2019 Sep). Monitoring breast cancer response to neoadjuvant chemotherapy using ultrasound strain elastography. Transl. Oncol..

[3] Elsayed B., Alksas A., Shehata M., Mahmoud A., Zaky M., Alghandour R. (2023 Nov). Exploring neoadjuvant chemotherapy, predictive models, radiomic, and pathological markers in breast cancer: a comprehensive review. Cancers (Basel).

[4] Agrawal S.K., Patel D., Shenoy P., Ahmed R., Arun I., Chatterjee S. (2023). Oncologic safety of breast conservation following NACT in women with locally advanced breast cancer. Ecancermedicalscience.

[5] Gulis K., Ellbrant J., Svensjö T., Skarping I., Vallon-Christersson J., Loman N. (2023). A prospective cohort study identifying radiologic and tumor related factors of importance for breast conserving surgery after neoadjuvant chemotherapy. Eur. J. Surg. Oncol. [Internet].

[6] Alberro J.A., Ballester B., Deulofeu P., Fabregas R., Fraile M., Gubern J.M. (2018 Jan 1). Long-term outcomes for neoadjuvant versus adjuvant chemotherapy in early breast cancer: meta-analysis of individual patient data from ten randomised trials. Lancet Oncol..

[7] Riedl O., Fitzal F., Mader N., Dubsky P., Rudas M., Mittlboeck M. (2009). Intraoperative frozen section analysis for breast-conserving therapy in 1016 patients with breast cancer. Eur. J. Surg. Oncol. [Internet].

[8] Schaefgen B., Funk A., Sinn H.P., Bruckner T., Gomez C., Harcos A. (2022 Feb). Does conventional specimen radiography after neoadjuvant chemotherapy of breast cancer help to reduce the rate of second surgeries?. Breast Cancer Res. Treat..

[9] Ramos M., Díez J.C., Ramos T., Ruano R., Sancho M., González-Orús J.M. (2014). Intraoperative ultrasound in conservative surgery for non-palpable breast cancer after neoadjuvant chemotherapy. Int. J. Surg..

[10] Bossuyt V., Provenzano E., Symmans W.F., Boughey J.C., Coles C., Curigliano G. (2015 Jul). Recommendations for standardized pathological characterization of residual disease for neoadjuvant clinical trials of breast cancer by the BIG-NABCG collaboration. Ann. Oncol. J. Eur. Soc. Med Oncol..

[11] Choi J., Laws A., Hu J., Barry W., Golshan M., King T. (2018 Nov). Margins in breast-conserving surgery after neoadjuvant therapy. Ann. Surg. Oncol..

[12] Kupstas A., Ibrar W., Hayward R.D., Ockner D., Wesen C., Falk J. (2018 Mar). A novel modality for intraoperative margin assessment and its impact on re-excision rates in breast conserving surgery. Am. J. Surg..

[13] Goh Y., Balasundaram G., Tan H.M., Putti T.C., Renzhe B., Hartman M. (2024). Ultrasound-guided photoacoustic (US-PA) tomography of the breast: Biochemical differentiation using intrinsic tissue markers—lipids, collagen and hemoglobin with histopathologic correlation. Sci. Rep. [Internet].

[14] Goh Y., Balasundaram G., Tan H.M., Putti T.C., Tang S.W., Ng C.W.Q. (2022 Sep). Biochemical “decoding” of breast ultrasound images with optoacoustic tomography fusion: First-in-human display of lipid and collagen signals on breast ultrasound. Photoacoustics.

[15] Balasundaram G., Goh Y., Moothanchery M., Attia A., Lim H.Q., Burton N.C. (2020 Sep). Optoacoustic characterization of breast conserving surgery specimens - a pilot study. Photoacoustics.

[16] Zhang J., Sun X., Li H., Ma H., Duan F., Wu Z. (2023 Apr). In vivo characterization and analysis of glioblastoma at different stages using multiscale photoacoustic molecular imaging. Photoacoustics.

[17] Zeng F., Fan Z., Li S., Li L., Sun T., Qiu Y. (2023 Oct). Tumor microenvironment activated photoacoustic-fluorescence bimodal nanoprobe for precise chemo-immunotherapy and immune response tracing of glioblastoma. ACS Nano.

[18] Matsumoto Y., Toi M., Toi M. (2023). Photoacoustic Imaging of Breast Cancer BT - Screening and Risk Reduction Strategies for Breast Cancer: Imaging Modality and Risk-Reduction Approaches.

[19] Toi M., Asao Y., Matsumoto Y., Sekiguchi H., Yoshikawa A., Takada M. (2017). Visualization of tumor-related blood vessels in human breast by photoacoustic imaging system with a hemispherical detector array. Sci. Rep. [Internet].

[20] Heijblom M., Piras D., Brinkhuis M., van Hespen J.C.G., van den Engh F.M., van der Schaaf M. (2015). Photoacoustic image patterns of breast carcinoma and comparisons with Magnetic Resonance Imaging and vascular stained histopathology. Sci. Rep. [Internet].

[21] Lin L., Hu P., Shi J., Appleton C.M., Maslov K., Li L. (2018 Jun). Single-breath-hold photoacoustic computed tomography of the breast. Nat. Commun..

[22] Goh Y., Balasundaram G., Tan H.M., Putti T.C., Ng C.W.Q., Fang E. (2022 Oct). Photoacoustic tomography appearance of fat necrosis: a first-in-human demonstration of biochemical signatures along with histological correlation. Diagn. (Basel, Switz. ).

[23] Neuschmelting V., Burton N.C., Lockau H., Urich A., Harmsen S., Ntziachristos V. (2016). Performance of a Multispectral Optoacoustic Tomography (MSOT) System equipped with 2D vs. 3D Handheld Probes for Potential Clinical Translation. Photoacoust. [Internet].

[24] Li G., Li L., Zhu L., Xia J., Wang L.V. (2015 Jun 1). Multiview Hilbert transformation for full-view photoacoustic computed tomography using a linear array. J. Biomed. Opt. [Internet].

[25] Wankhade D., Gharde P., Dutta S. (2023 Nov). The current role of neoadjuvant chemotherapy in the management of HER2-positive, triple-negative, and micropapillary breast cancer: a narrative review. Cureus.

[26] Leon-Ferre R.A., Hieken T.J., Boughey J.C. (2021 Apr). The landmark series: neoadjuvant chemotherapy for triple-negative and HER2-positive breast cancer. Ann. Surg. Oncol..

[27] Rossou C., Alampritis G., Patel B. (2024 Jan 1). Reducing re-excision rates in breast conserving surgery with Margin Probe: systematic review. Br. J. Surg. [Internet].

[28] Gole L., Yeong J., Lim J.C.T., Ong K.H., Han H., Thike A.A. (2020). Quantitative stain-free imaging and digital profiling of collagen structure reveal diverse survival of triple negative breast cancer patients. Breast Cancer Res [Internet].

[29] Mercep E., Dean-Ben X.L., Razansky D. (2017 Oct). Combined pulse-echo ultrasound and multispectral optoacoustic tomography with a multi-segment detector array. IEEE Trans. Med Imaging.

[30] Manwar R., Lara J.B., Prakash R., Ranjbaran S.M., Avanaki K. (2022 Jun). Randomized multi-angle illumination for improved linear array photoacoustic computed tomography in brain. J. Biophoton..

[31] Tzoumas S., Nunes A., Olefir I., Stangl S., Symvoulidis P., Glasl S. (2016). Eigenspectra optoacoustic tomography achieves quantitative blood oxygenation imaging deep in tissues. Nat. Commun. [Internet].

[32] Liu Y., Yuan Z. (2016). Biomedical Optics 2016 [Internet].

