# Supplementary material for: Utility of photoacoustic patterns in intra-operative margin assessment of breast cancer post neoadjuvant chemotherapy
Source: Photoacoustics. 2025 Feb 23;43:100701. doi: 10.1016/j.pacs.2025.100701 (PMC11964573; doi:10.1016/j.pacs.2025.100701)
Supplement: Supplementary file 1 — Supplementary material [file mmc1.docx]

**Supplementary information**

**Utility of photoacoustic patterns in intra-operative margin assessment of breast cancer post neoadjuvant chemotherapy**

**Authors**

Yonggeng Goh^#1,^ Ghayathri Balasundaram^#2,^ Hui Min Tan^#3^, Thomas Choudary Putti^3^, Bi Renzhe^2^, Mikael Hartman^4^, Shaik Ahmad Buhari^4^, Celene Wei Qi Ng^4^, Su Ann Lui^4^, Serene Si Ning Goh^4^, Wei Qi Leong^4^, Eric Fang^1^, Malini Olivo^2^*, Quek Swee Tian^1^*

**Affiliations**

^1^Department of Diagnostic Imaging, National University Hospital, 5 Lower Kent Ridge Road, Singapore 119074

^2^A*STAR Skin Research Labs, Agency for Science, Technology and Research (A*STAR), 31 Biopolis Way, #07-01, Nanos, Singapore 138669

^3^ Department of Pathology, National University Hospital, 5 Lower Kent Ridge Road, Singapore 119074

^4^Department of Breast Surgery, National University Hospital, 5 Lower Kent Ridge Road, Singapore 119074

**Corresponding authors*

*^#^These authors contributed equally to this work*

.

**Equipment and Imaging Protocol**


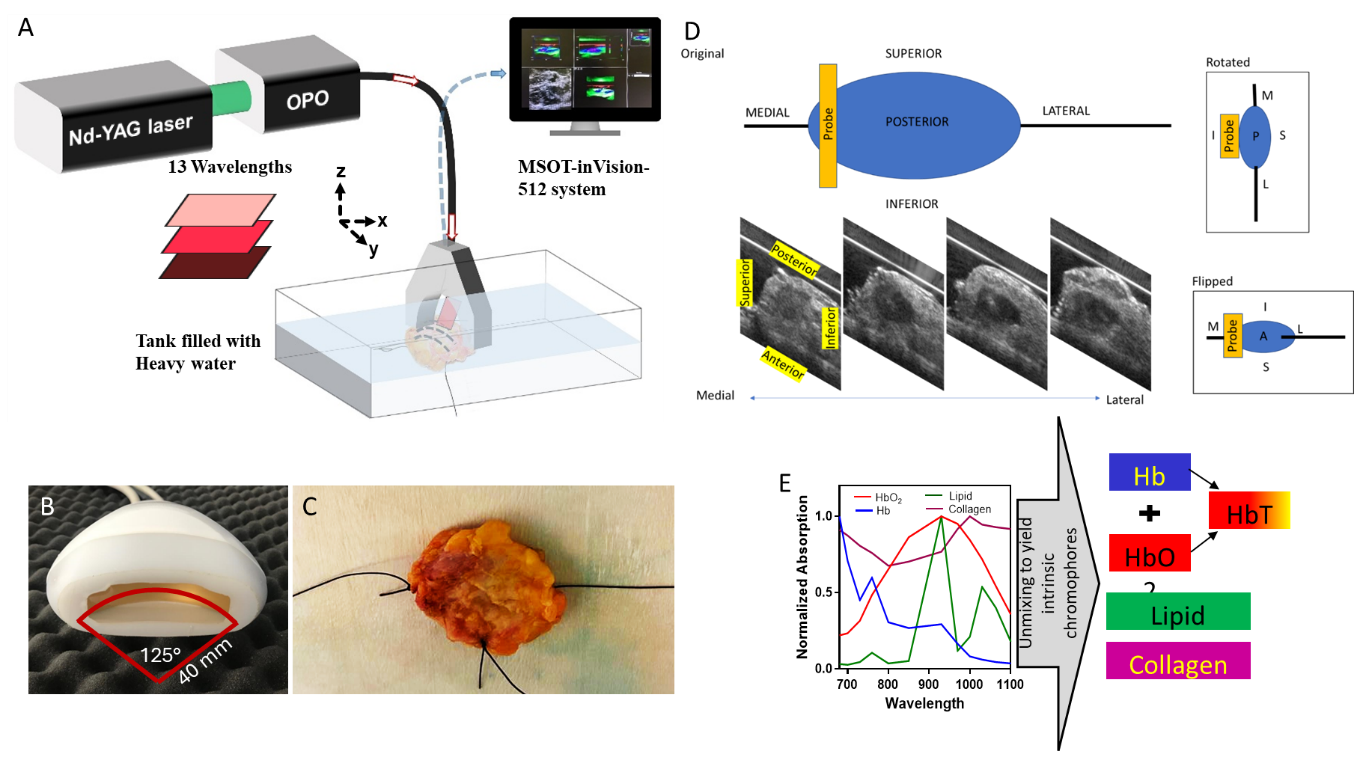


***Fig S1.*** *US-PA specimen imaging setup (A) Schematic of the handheld US-OT probe placed in heavy water tank on the sample. (B) Photograph of the 2D array curvilinear US-OT probe that has 256 detector elements arranged along an angle of 125° and radius of 40 mm. (C) Freshly excised breast cancer specimen with margins labelled using silk stitches - Long stitch: Lateral margin, Medium length stitch: Medial margin, Short stitch: Superior margin, Loop stitch: Anterior margin.(D) Schematic of the specimen showing the different margins based on the silk thread in original positioning and the sections of ultrasound images acquired from medial to lateral margins with schematic of the specimen in rotated and flipped positions, respectively. (E) Normalized absorption spectra of endogenous chromophores in excised breast specimens from 700 – 1100 nm based on which the acquired acoustic signals were unmixed to yield distribution maps of HbT (Hb+HbO_2_), collagen and lipid.*

After breast tissue was excised following lumpectomy, the specimen was oriented using silk stitches by the surgeons to maintain the original anatomical reference points, as depicted in **Figure S1**. The tissue was then subjected to a saline rinse to remove any residual surface blood, ensuring clear imaging of subsurface structures. All tissue specimens used in this ex vivo study were collected within 10 minutes after surgery in the operating theatre, and promptly imaged to preserve the integrity of blood signals.

For the ultrasound-photoacoustic (US-PA) imaging, we employed the **MSOT inVision 512-ECHO system**  (iThera Medical GmbH, Munich, Germany) (Fig S1.A), which was coupled with a custom-designed handheld two-dimensional (2D) optoacoustic probe (Fig S1.B). The probe featured an arc-shaped array comprising 256 detector elements arranged on a spherical surface with a 40 mm radius, providing 125º of angular coverage. These detector elements operated at a central frequency of 5 MHz, which was optimized for photoacoustic and ultrasound signal acquisition in soft tissue, such as breast tissue.

The breast tissue specimen with margins labelled using silk stitches (Fig S1.C) was carefully placed on a specially designed imaging platform, capable of multidirectional movement, including horizontal, vertical, and rotational motions, allowing for comprehensive imaging coverage. When the flip side of the tissue needed to be imaged, the specimen was manually flipped by the operator. Scanning commenced by positioning the handheld probe on a computer-controlled stage, which started from the thickest part of the tissue and moved incrementally both horizontally and vertically across the specimen (Fig S1.D). To ensure optimal acoustic coupling and prevent the presence of air pockets, the tissue was placed on a silicone bed submerged within an imaging chamber filled with heavy water (**D_2_O**).

The handheld probe scanned the entire specimen in fine increments of 1 mm, enabling high-resolution capture of photoacoustic signals from the tissue. Light delivery was achieved using a fiber optic bundle integrated into the handheld probe, which was connected to a wavelength-tunable optical parametric oscillator (OPO). The OPO could deliver light in a tunable wavelength range between 660 nm and 1300 nm, at a repetition rate of 10 Hz, with a per-pulse energy of 80 mJ at 730 nm. Multiple wavelengths (ranging from 700 nm to 1100 nm) were sequentially used to acquire data from light-absorbing chromophores within the breast tissue, including deoxyhemoglobin (Hb), oxyhemoglobin (HbO2), lipids, and collagen. The applied fluence during imaging was kept below 20 mJ/cm², adhering to the safety standards for near-infrared (NIR) nanosecond laser exposure set by the American National Standards Institute (ANSI).

Throughout the imaging process, real-time images were reconstructed using a back-projection algorithm and displayed on the system monitor. These images allowed for live monitoring and data validation as the probe scanned through the specimen. Upon completion of US-PA imaging, the tissue specimens were immersed in formalin and transported to the histopathology laboratory for further examination. Offline reconstruction of images acquired at each wavelength was conducted using default settings (back-projection algorithm, cut-off frequencies at 0.5 khz to 6.5 Mhz) on ViewMSOT 3.8. Spectral unmixing was performed using the default linear regression algorithm in ViewMSOT 3.8 to differentiate between chromophores, including Hb, HbO2, lipids, and collagen (Fig S1.E). Collagen and lipid signals were unmixed based on the entire spectral range (700 – 1100 nm), while Hb and HbO2 signals were calculated from a sub-range (700-850 nm) for enhanced accuracy in unmixing due to lower water absorptivity at these wavelengths. Color maps were assigned to each chromophore: lipids were represented in green, collagen in magenta, and total hemoglobin (HbT) in red.

**Histopathological Examination and Correlation with PA Imaging**

After the US-PA imaging, the plane of image acquisition was precisely communicated to the pathologist, who sectioned the specimen accordingly to ensure alignment with the imaging plane for accurate histopathological correlation. Histological sections were obtained from the same regions of interest (ROIs) identified during photoacoustic imaging. The primary objective was to correlate features observed in photoacoustic images—such as suspicious lesions, residual tumors, and tumor margins—with the histological findings.

Next, a team of PA scientists, radiologist and pathologist would sit down together in a multi-disciplinary meeting to orientate the post processed PA and histology images together to ascertain its accuracy. During the meeting, the accuracy is confirmed by identifying the primary index mass/cancer or the localization clip (in post-chemotherapy cases) and its relationship with adjacent margins. Once the histopathological slides were processed, a multidisciplinary meeting was held involving a radiologist, a photoacoustic scientist, and a pathologist. In this meeting, the team collaboratively reviewed both the photoacoustic images and the histopathological slides to identify the index or suspicious lesions and assess their relationship to the surrounding margins. This correlation was crucial for evaluating the accuracy of photoacoustic imaging in detecting tumor presence and assessing the proximity of cancerous tissue to surgical margins.

For patients who exhibited a complete response to neoadjuvant therapy, the metallic clip inserted at the tumor site during the initial biopsy was used as a reference point to locate the center of the original lesion. In cases where patients had no response or only partial response to therapy, the residual tumor itself was used as the region of interest for imaging and histological correlation. This systematic comparison between photoacoustic imaging and histopathology helped validate the utility of US-PA technology for accurate tumor margin assessment in lumpectomy specimens, potentially guiding future clinical use for breast-conserving surgeries.
